# Differential Characteristics Based Iterative Multiuser Detection for Wireless Sensor Networks

**DOI:** 10.3390/s17020388

**Published:** 2017-02-16

**Authors:** Xiaoguang Chen, Xu Jiang, Zhilu Wu, Shufeng Zhuang

**Affiliations:** 1 School of Astronautics, Harbin Institute of Technology, Harbin 150001, China; chen_xiaoguang@hotmail.com; 2 China Academy of Space Technology, Beijing 100089, China; 3 School of Electronics and Information Engineering, Harbin Institute of Technology, Harbin 150001, China; jiang520410@126.com (X.J.); zhuangshufeng@163.com (S.Z.)

**Keywords:** iterative multiuser detection, differential characteristics, threshold detection, error bit correction

## Abstract

High throughput, low latency and reliable communication has always been a hot topic for wireless sensor networks (WSNs) in various applications. Multiuser detection is widely used to suppress the bad effect of multiple access interference in WSNs. In this paper, a novel multiuser detection method based on differential characteristics is proposed to suppress multiple access interference. The proposed iterative receive method consists of three stages. Firstly, a differential characteristics function is presented based on the optimal multiuser detection decision function; then on the basis of differential characteristics, a preliminary threshold detection is utilized to find the potential wrongly received bits; after that an error bit corrector is employed to correct the wrong bits. In order to further lower the bit error ratio (BER), the differential characteristics calculation, threshold detection and error bit correction process described above are iteratively executed. Simulation results show that after only a few iterations the proposed multiuser detection method can achieve satisfactory BER performance. Besides, BER and near far resistance performance are much better than traditional suboptimal multiuser detection methods. Furthermore, the proposed iterative multiuser detection method also has a large system capacity.

## 1. Introduction

Wireless sensor network (WSN) is a special kind of ad-hoc network which can be applied in military, home industrial and other monitoring scenarios [1]. High throughput and low latency sensor networks ubiquitously provide connections around us. Most wireless sensor networks focus on narrowband physical layers such as IEEE 802.15.4 on 2.4 GHz. However, ultra-wideband (UWB) technology provides high data transmission rates, strong multipath resolution and high interference resistance ability which is more suitable for communication links or WSNs in specific scenarios such as multimedia transmission, dense multipath scenarios and high reliability communication.

In recent years, WSN based on UWB has been explored in various scenarios including wireless monitoring sensor networks in underground mine tunnels [2], smart buildings [3], marine engine telemetry sensor networks [4], aircraft sensor networks [5] and wireless body sensor networks [6]. For star topology wireless sensor networks based on UWB, direct sequence UWB (DS-UWB) is a suitable technology to provide reliable multiple access for each sensor node. In a sense, the multiple access scheme of DS-UWB is similar to code division multiple access (CDMA) systems, as both of them use pseudorandom codes to distinguish different users. For such a DS-UWB system, the multiple access interference and noise are serious limiting conditions for improving its bit error ratio (BER) performance.

Multiuser detection is an effective method to eliminate the bad effect of multiple access interference. The optimal multiuser detection (OMUD) method was first proposed by Verdu in 1986 [7]. With a bank of matched filters followed by a maximum likelihood sequence estimator, optimal multiuser detection achieves optimal BER and near-far effect resistance performances. However, optimal multiuser detection has a computational complexity that grows exponentially with the number of users. This feature makes optimal multiuser detection impractical to implement in actual communication systems.

Consequently, many studies have been performed on suboptimal multiuser to obtain less complexity and good BER performance. Of them, linear multiuser detection has been widely studied as low-complexity integrated mathematical analysis [8,9]. Matched filter (MF), decorrelating (DEC) and minimum mean square error (MMSE) are three representative linear multiuser detection methods. Linear multiuser detectors form the output based on various linear filters. In recent years, considerable attention has been focused on blind multiuser detection, which requires the prior knowledge of only the signature sequence and timing of the desired user [10,11,12,13,14,15,16]. In [13], a novel subspace tracking algorithm by orthonormalizing the eigenvectors using an approximation of Gram-Schmidt procedure was applied to blind multiuser detection. This scheme demonstrates the performance improvement for non-stationary cellular CDMA environment. Besides, suboptimal multiuser detection algorithms based on SINR Lower Bound [17], box-constrained deregularization [18], compressive sensing [19], restricted search space [20], decision-level data fusion technologies [21] and opportunistic multiuser detection [22,23] were analyzed in recent years. As for coded DS-UWB systems, iterative multiuser detection methods were also widely analyzed. Usually these suboptimal multiuser detection algorithms seek tradeoff between computation complexity and BER performance. On the basis of the Turbo decoding algorithm, various iterative multiuser detection algorithm were proposed [24,25,26,27]. Reference [26] proposed an iterative multiuser detector for overloaded LDPC coded CDMA systems. This receiver consists of a combination of matched filters in the first stage and a linear MMSE detector and an interference cancellation scheme in the successive stages. In this way, the receiver achieved better BER performance than Turbo coded systems.

In this paper, an iterative multiuser detection algorithm based on differential characteristics is proposed for DS-UWB systems. On the basis of optimal multiuser detection, differential characteristics are derived to map the received bit sequence in to a one-dimensional feature space, So that the wrongly received bits are easy to distinguish. Then threshold detection is utilized to get a preliminary detection of the potential wrongly received bit because the optimal threshold is difficult to compute. After that an error bit correct procedure is employed to correct the mistakes in the previous preliminary threshold detection. The above described differential characteristics calculation, preliminary threshold detection and error bit correction procedures are iterated to achieve better EBR performance. Simulation results demonstrate performance improvement compared to traditional suboptimal multiuser detection methods.

The reminder of this paper is organized as follows: Section 2 introduces the system model DS-UWB system and several typical multiuser detection algorithms. In Section 3, the proposed multiuser detection method is described in detail. Simulations and discussions are given in Section 4. Conclusions are in Section 5.

## 2. Several Multiuser Detection Methods for DS-UWB Systems

Suppose *K* users transmit signals simultaneously in a DS-UWB system. In this binary phase shift key (BPSK) modulated DS-UWB system, the received signal can be expressed as:

(1) $$r\left(t\right)=\sum_{k=1}^{K}\sqrt{E_{k}}S_{k}\left(t\right)d_{k}+n\left(t\right)\;0\leq n\leq T$$

where *E_k_* denotes the bit energy of the *k*th user; *S_k_*(*t*) denotes the unit-energy signature waveform which is constructed by the pseudo random sequences of the *k*th user; $d_{k}\in \left\{+1,\;-1\right\}$ denotes the bit value of *k*th user; *n*(*t*) denotes the noise with power spectral density *N*_0_, and *T* denotes the bit interval.

After a bank of matched filters, the output of *k*th user sampled at *T* is achieved by the following expression:

(2) $$y_{k}=\int_{0}^{T}r\left(t\right)S_{k}\left(t\right)dt=\sqrt{E_{k}}d_{k}+\sum_{\begin{array}{l} j=1\\ j\neq k \end{array}}^{K}\sqrt{E_{j}}\rho_{jk}d_{j}+n_{k}$$

where the self-correlation of user *k* is normalized to 1, i.e., $\rho_{kk}=\int_{0}^{T}S_{k}\left(t\right)S_{k}\left(t\right)dt=1$, the cross correlation of user *j* and *k* is $\rho_{jk}=\int_{0}^{T}S_{j}\left(t\right)S_{k}\left(t\right)dt$, and the noise at the output of *k*th user is $n_{k}=\int_{0}^{T}S_{k}\left(t\right)n\left(t\right)dt$. In principle, it is assumed that $\rho_{ii}\gg \rho_{ij},\;i\neq j$.

The matched filter outputs of the *K* users can be written in vector form as:

(3) $$y={\left[y_{1}\;y_{2}\cdots y_{K}\right]}^{T}=RAd+n$$

where **R** is the correlation matrix of the signature waveforms, i.e., $R_{ij}=\rho_{ij}$, $A=\mathrm{diag}\left[\sqrt{E_{1}}\;\sqrt{E_{2}}\cdots \sqrt{E_{K}}\right]$, $d=\left[d_{1}\;d_{2}\cdots d_{K}\right]$, and **n** is the *K* × 1 zero-mean additive white Gaussian noise vector.

The symbol decisions after matched filters are given by:

(4) b=sgn(y)

As is shown in (2), each element of **b** is interfered by other *K*-1 elements of **d** due to $R_{ij}\neq 0\left(\mathrm{when}\;i\neq j\right)$. Thus various multiuser detection algorithms were proposed to eliminate the bad effect of multiple access interference.

On the basis of matched filter, optimal multiuser detection takes advantages of maximum likelihood sequence estimation algorithm. The likelihood function can be expressed as:

(5) $$p(y|b)=\exp(\frac{-\frac{1}{2}{\left(y-RAb\right)}^{T}{\left(\sigma^{2}R\right)}^{-1}(y-RAb)}{{\left(2\pi \right)}^{K/2}\sigma |R{|}^{1/2}})$$

where **|R|** denotes the determinant of **R**. the maximum likelihood symbol decisions can be written as:

(6) $$b_{\mathrm{OMD}}=\arg\;\underset{b}{\max}\{2b^{T}Ay-b^{T}ARAb\}$$

The above maximization problem is a combinatorial optimization problem which is known to be NP-hard: its computational complexity increases exponentially with the number of user in a WSN. This *O*(2*^K^*) implementation complexity required by optimal multiuser detection makes it impractical for real system. Consequently, various low complexity multiuser detection algorithms are proposed to achieve the tradeoff problem between computational complexity and BER performance. Optimal multiuser detection represents, however, a basis for comparison for other suboptimal detectors.

Linear multiuser detection is an important class of low complexity multiuser detection algorithms. Linear multiuser detectors form the output **b***_linear_* based on various linear filters. The output of linear multiuser detectors follows the following expressions:

(7) $$b_{linear}=\mathrm{sgn}\left(My\right)$$

For decorrelating multiuser detection we can get:

(8) $$M=R^{-1}$$

For the minimum mean square error (MMSE) multiuser detection we obtain:

(9) $$M=A^{-1}{\left(R+\sigma^{2}A^{2}\right)}^{-1}$$

For decorrelating a multiuser detector the output is interference free, but the background noise is enhanced by the transformation **R**^−1^, while MMSE detection balances the desire to completely eliminate the multiple access interference with the desire of avoiding the background noise enhancement.

## 3. Multiuser Detection Based on Differential Characteristics

**Figure 1:**
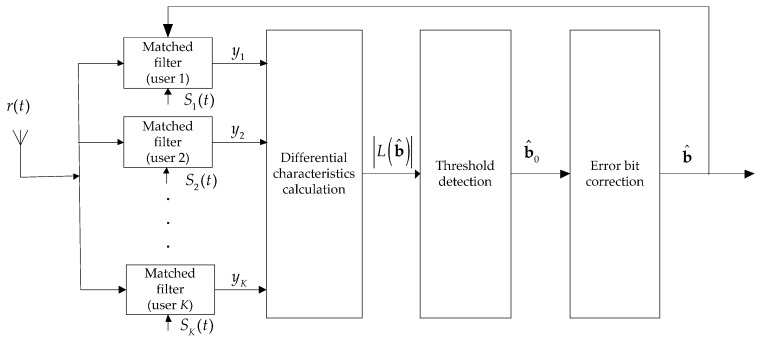
Diagram of the proposed multiuser detection algorithm.

The main idea of the proposed multiuser detection method is to iteratively find the error bits from the received signals and correct them. The block diagram of the proposed iterative multiuser detection algorithm is shown below in Figure 1. Firstly, the differential characteristics are calculated and the outputs of matched filters are mapped in to a one dimension space due to optimal multiuser detection is a multi-dimensional optimization problem; then a primary threshold classification is conducted to find the potential incorrectly received bits; after that an error bit corrector is employed to correct the wrong bits. The three stage process above is iterative executed to further lower the BER.

### 3.1. Differential Characteristics Calculation

On the basis of the decision function of optimal multiuser detection, let:

(10) $$F(b)=\frac{1}{2}b^{T}ARAb-b^{T}Ay=\frac{1}{2}\sum_{i=1}^{K}\sum_{j=1}^{K}A_{i}A_{j}\rho_{ij}b_{i}b_{j}-\sum_{i=1}^{K}b_{i}A_{i}y_{i}$$

From (10), it is clear that function *F*(**b**) is a quadratic form of vector **b**, which means this is a non-linear equation with the order of two. And it is not easy to solve the minimum of *F*(**b**) and the corresponding value of **b**. In addition, the function *F*(**b**) embraces cross-component *b_i_b_j_* (*i*, *j* = 1, 2,…, *K*, *i* ≠ *j*), and we can’t judge a bit independently by *F*(**b**). Hence, it is not appropriate for function *F*(**b**) to be the characteristic function.

In this case, it is better to calculate the derivative of function *F*(**b**) to decrease the order of characteristic function. Calculating the partial derivation of (10), we get:

(11) $$\frac{\partial F}{\partial b}=ARAb-Ay$$

By expanding (11), we get the *K*th-order linear equations given as follows:

(12) $$\{\begin{array}{c} \frac{\partial F}{\partial b_{1}}=\sum_{j=1}^{K}A_{1}A_{j}\rho_{1j}b_{j}-A_{1}y_{1}\\ \frac{\partial F}{\partial b_{2}}=\sum_{j=1}^{K}A_{2}A_{j}\rho_{2j}b_{j}-A_{2}y_{2}\\ ...\\ \frac{\partial F}{\partial b_{K}}=\sum_{j=1}^{K}A_{K}A_{j}\rho_{Kj}b_{j}-A_{K}y_{K} \end{array}$$

Because *F*(**b**) is a discrete equation, we cannot just setting the partial derivative described above equal to 0 and get the stationary point of *F*(**b**). Let $L(b_{i})=\sum_{j=1}^{K}A_{i}A_{j}\rho_{ij}b_{j}-A_{i}y_{i}$, *i* = 1, 2, …, *K*. Substituting the candidate sequence **b** into (12) (Results of conventional suboptimal multiuser detectors can be employed as the candidate sequence **b**. In this paper, the output of matched filter as shown in (4) is employed.), there will be two situations as discussed below:

1. No wrong bit in **b**

Based on the theory of extreme value, if multiple access interference is the only interference resource without AWGN and the elements in **b** are all correct, the result of (12) strictly equals 0. Substituting (3) into *L*(*b_k_*), we can see that:

(13) $$L\left(b_{k}\right)=-A_{k}n_{k}\sim N\left(0,\;A_{i}^{2}N_{0}^{2}/2\right)\;k=1,2,\cdots ,K$$

Apparently, *L*(*b_k_*) is a zero-mean Gaussian random variable.

2. Wrong bit exists in **b**

Suppose *b_i_* (*i* =1, 2,…, *K*) is the wrongly received bit (in other words, −*b_i_* is the correct bit), the other bits are correct. Substituting it to the *i*th equation of (12), we get:

(14) $$\begin{array}{ll} L(b_{i}) & =\sum_{\begin{array}{l} j=1\\ j\neq i \end{array}}^{K}A_{i}A_{j}\rho_{ij}b_{j}-A_{i}y_{i}+A_{i}^{2}\rho_{ii}b_{i}=\sum_{\begin{array}{l} j=1\\ j\neq i \end{array}}^{K}A_{i}A_{j}\rho_{ij}b_{j}-A_{i}y_{i}+A_{i}^{2}\rho_{ii}(-b_{i})+2A_{i}^{2}\rho_{ii}b_{i}\\ & =2A_{i}^{2}N_{c}b_{i}-A_{i}n_{i} \end{array}$$

As for *k* which is not equal to *i*, we get:

(15) $$L(b_{k})=2A_{i}A_{k}\rho_{ik}b_{i}-A_{k}n_{k}\;k=1,2,\cdots ,K\;k\neq i.$$

From the above, we can get:

(16) $$L(b_{i})\sim N(2A_{i}^{2}b_{i},\frac{A_{i}^{2}N_{0}}{2})$$

(17) $$L(b_{k})\sim N(2A_{i}A_{k}\rho_{ik}b_{k},\frac{A_{k}^{2}N_{0}}{2})\;k=1,2,\cdots ,K\;k\neq i$$

According to (16) and (17), we can see when the *i*th bit of **b** is wrong, then we get |*L*(*b_i_*)| >> |*L*(*b_k_*)|, *k* = 1, 2, …, *K*, *k* ≠ *i* with a large probability. Therefore, the function *L*(**b**) can obviously differentiate the wrong bits and the right bits through the absolute value of it. In addition, *L*(**b**) is a *K*th-order linear equations which can get the result of *L*(**b**) without complex computations. In conclusion, it is appropriate to set |*L*(**b**)| as the differential characteristics function in the multiuser detection algorithm.

**Figure 2:**
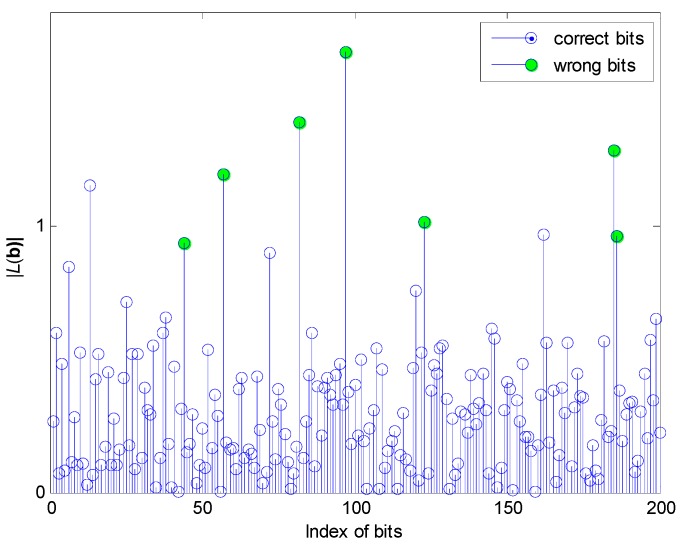
The value of differential characteristics function |*L*(*b_k_*)|.

Here is an example of the proposed differential characteristics function |*L*(**b**)| with a condition of 10 users, 6 dB of receiver’s output signal-to-noise ratio (SNR), spread spectrum gain *N_c_* = 63, which is shown in Figure 2.

According to Figure 2, it is clear that most of the wrong bits and the right ones have a significant difference in the feature space mapped by |*L*(**b**)| among the 200 tested bits, so it is accessible to map **b** into a one-dimension feature space |*L*(**b**)| to preliminarily identify the wrongly received bits.

### 3.2. Preliminary Threshold Detection

As is described in the above Section 3.1, it is feasible to distinguish the wrongly received bits by threshold detection. Set two statuses *H*_0_ and *H*_1_, *H*_0_ represents the status where candidate bit *b_k_* is correct, and *H*_1_ represents the status where candidate bit *b_k_* is wrong. Let *Z_k_* = |*L*(*b_k_*)|, according to $\rho_{ik}\gg \rho_{ik},\;i\neq k$, we can see that the probability density functions of *Z_k_* in *H*_0_ and *H*_1_ are given as follows:

(18) $$H_{0}:f_{Z_{k}}(y)=\{\begin{array}{cc} 0 & y\leq 0\\ \frac{2}{A\sqrt{\pi N_{0}}}\exp[-\frac{y^{2}}{A^{2}N_{0}}] & y>0 \end{array}$$

(19) $$H_{1}:f_{Z_{k}}(y)=\{\begin{array}{cc} 0 & y\leq 0\\ \frac{1}{A\sqrt{\pi N_{0}}}\{\exp[-\frac{{\left(y-2A^{2}\right)}^{2}}{A^{2}N_{0}}]+\exp[-\frac{{\left(y+2A^{2}\right)}^{2}}{A^{2}N_{0}}]\} & y>0 \end{array}$$

**Figure 3:**
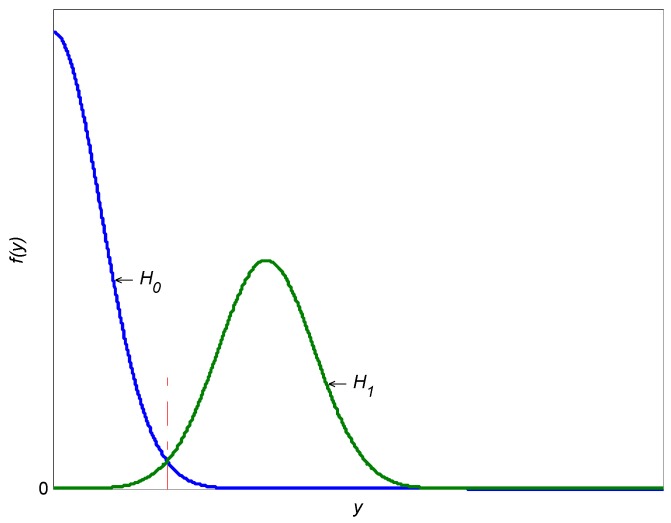
The probability density functions of two statuses.

The curves of the probability density functions in the two status are depicted in Figure 3. The red dashed line shows the optimal threshold.

Set *a* as the judgment threshold. If *Z_k_* > *a*, verdict *b_k_* is the wrong bit; or else, verdict *b_k_* is the correct bit. The probability of detection is given as

(20) $$P(Z_{k}>a|H_{1})=\frac{1}{2}[erfc(\frac{a-2A^{2}}{A\sqrt{N_{0}}})+erfc(\frac{a+2A^{2}}{A\sqrt{N_{0}}})]$$

The probability of false alarm is given as

(21) $$P(Z_{k}>a|H_{0})=erfc(\frac{a}{A\sqrt{N_{0}}})$$

Set *P_e_* as the average BER of candidate bits, according to Equations (19) and (20), the BER after threshold detection equals:

(22) $$P_{e,out}(a)=\frac{1}{2}P_{e}[erfc(\frac{2A^{2}-a}{A\sqrt{N_{0}}})+erfc(\frac{2A^{2}+a}{A\sqrt{N_{0}}})]+(1-P_{e})erfc(\frac{a}{A\sqrt{N_{0}}})$$

Therefore we can adjust the value of the threshold to minimize the value of *P_e,out_*, the value of *a*, which corresponds to the minimum, is called the optimal threshold. However, the optimal threshold is relevant to many factors, including SNR and the BER of the candidate bit set. Hence, it’s difficult to set an accurate threshold to obtain the best judgment in different situations.

Therefore, a suboptimal threshold is selected to get the preliminary wrong bit recognition. From (19) we can see that when *y* > 0, the second term of $f_{z_{k}}\left(y\right)$ is approximately equal to zero, especially at high SNR, i.e., noise power spectral density *N*_0_ is small compared to *A*, so the second term of $f_{z_{k}}\left(y\right)$ can be ignored. For this approximation, according to (18) and (19) the solution of the following equation can be regarded as the best threshold:

(23) $$\frac{2}{A\sqrt{\pi N_{0}}}\exp\left(-\frac{y^{2}}{A^{2}N_{0}}\right)=\frac{1}{A\sqrt{\pi N_{0}}}\exp\left(-\frac{{\left(y-2A^{2}\right)}^{2}}{A^{2}N_{0}}\right),\;y>0$$

Solving this equation we can get:

(24) $$\hat{y}=A^{2}+\frac{\ln\left(2\right)}{2}N_{0}\approx A^{2}+0.3466N_{0}$$

From the above discussion, the calculation of threshold described in (24) needs accurate estimation of noise power spectral density *N*_0_. Because $A^{2}$ is larger than *N*_0_ in actual communication system when SNR > 0 (in dB), thus the second term in (24) can be ignored. In this paper we take $A_{i}^{2}$ as the detection threshold of the *i*th received bit. Furthermore, lower threshold results in higher detection probability, at the cost of higher false alarm probability. Then the next stage, error bit correction, will be utilized to reduce the false alarm probability.

### 3.3. Error Bit Correction

In Section 3.2 we recognize the wrong bits only by the absolute value of *L*(**b**), which sometimes may regard some correct bits as wrong bits and decrease the BER performance of the system. Section 3.2 cannot get the optimal threshold, therefore further processing method need to be taken to lower BER.

As analyzed in Section 3.1, when the *i*th bit of b is wrong, from (16) and (17) we get:

(25) $$L(b_{i})\sim N(2A_{i}^{2}b_{i},\frac{A_{i}^{2}N_{0}}{2})$$

(26) $$L(b_{k})\sim N(2A_{i}A_{k}\rho_{ik}b_{k},\frac{A_{k}^{2}N_{0}}{2})\;k=1,2,\cdots ,K\;k\neq i$$

Let *f*(*x*) and *g*(*x*) be the probability density function of |*L*(*b_i_*)| and |*L*(*b_k_*)|, respectively. We can get that:

(27) $$f\left(x\right)=\{\begin{array}{cc} 0 & x\leq 0\\ \frac{1}{A_{i}\sqrt{\pi N_{0}}}\left\{\exp\left[-\frac{{\left(x-2A_{i}^{2}\right)}^{2}}{A_{i}^{2}N_{0}}\right]+\exp\left[-\frac{{\left(x+2A_{i}^{2}\right)}^{2}}{A_{i}^{2}N_{0}}\right]\right\} & x>0 \end{array}$$

(28) $$g\left(x\right)=\{\begin{array}{cc} 0 & x\leq 0\\ \frac{1}{A_{k}\sqrt{\pi N_{0}}}\left\{\exp\left[-\frac{{\left(x-2A_{i}A_{k}\rho_{ik}\right)}^{2}}{A_{k}^{2}N_{0}}\right]+\exp\left[-\frac{{\left(x+2A_{i}A_{k}\rho_{ik}\right)}^{2}}{A_{k}^{2}N_{0}}\right]\right\} & x>0 \end{array}$$

Similar as (23), let *f*(*x*) = *g*(*x*), we can get the optimal threshold *x*_0_. And we can get the probability that |*L*(*b_i_*)| >> |*L*(*b_k_*)|:

(29) $$P\left(\left|L\left(b_{i}\right)\right|>\left|L\left(b_{k}\right)\right|\right)=\int_{x_{0}}^{+\infty }\left[f\left(x\right)-g(x)\right]dx$$

**Figure 4:**
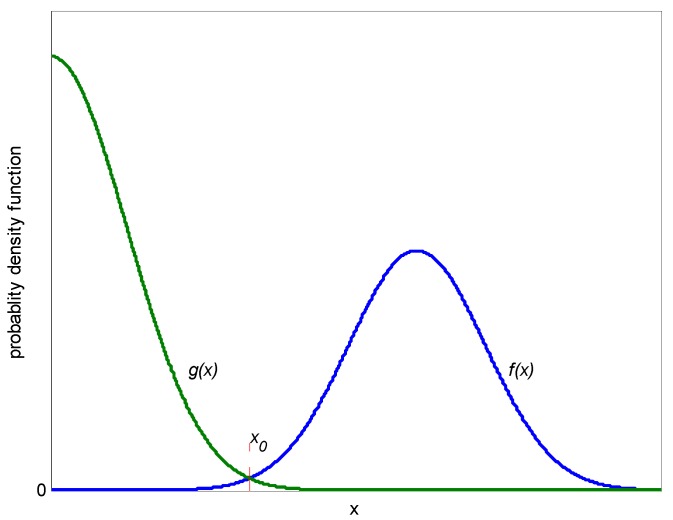
The probability density function curves of |*L*(*b_i_*)| and |*L*(*b_k_*)|.

However, from (27) and (28) we find that the optimal *x*_0_ is not easy to calculate. Besides, the integral in (29) is hard to calculate even when we get the accurate value of *x*_0_. As is known 1 >> $\rho_{ij},\;\left(i\neq j\right)$, the probability density function curves of |*L*(*b_i_*)| and |*L*(*b_k_*)| is shown in Figure 4.

From (29) and Figure 4 we can conclude that |*L*(*b_i_*)| >> |*L*(*b_k_*)|, *k* = 1, 2,…, *K*, *k* ≠ *i* with a large probability. Thus the bits which are recognized as wrongly received in Section 3.2 can be further detected. When the i*th* received bit *b_i_* is judged as wrongly received bit in Section 3.2, we can calculate |*L*(−*b_i_*)|. If |*L*(−*b_i_*)| > |*L*(*b_i_*)|,we conclude that the value of *i*th bit is *b_i_*, else we conclude that the value of *i*th bit is −*b_i_*.

After the above three stage process procedure, there may still exists wrongly judged bits. Thus the differential characteristics calculation, threshold detection and error bit correction process are iterated as shown in Figure 1 to further lower the BER.

Now we discuss the computational complexity of the proposed multiuser detection algorithm. From (11) and (12), we can conclude that the computational complexity of differential characteristics function |*L*(**b**)| is linear to number of users *K*. The second stage is threshold detection, in which only *K* comparisons are needed. The third stage recalculates the differential characteristics function for those potential wrongly received bits, so this computational complexity of this stage is much lower than the first stage, especially at high SNR. For this proposed iterative receiver, the total computational complexity is then linear to *nK*, where *n* is the number of iterations and *K* is the number of users.

## 4. Simulation Results and Discussions

In this section we demonstrate the simulation results for star topology UWB based wireless sensor network. In this network each sensor node communicate with the sink node with a specific pseudo random sequence, i.e., the network employ the DS-UWB multiple access strategy. The performance of five multiuser detection strategies are presented here, including matched filter (MF), decorrlating detector (DEC), minimum mean square error (MMSE), optimal detector (OMUD) and the proposed iterative detector based on differential characteristics.

During the simulation in this section the signal is transmitted over 3–6 GHz and the chip transmission rate is 2 GHz. Two types of channel are simulated in this section, one is the AWGN channel, the other is a multipath channel, the IEEE 802.15.4a channel (without loss of generality, IEEE 802.15.4a CM1 channel is studied for simulation). For the AWGN channel the signal is received with simple matched filter, while for the multipath IEEE 82.15.4a channel we choose the selective RAKE (S-RAKE) receiver. The S-RAKE receiver chooses 16 branches and uses the maximum ratio combining (MRC) rule. Our main object in this section is to check the BER performance of the proposed iterative multiuser detector based on differential characteristics. Monte Carlo simulations are utilized to examine the proposed iterative multiuser detection algorithm in this section. Major simulation parameters employed in this section are listed in Table 1.

### 4.1. Number of Iterations

**Figure 5:**
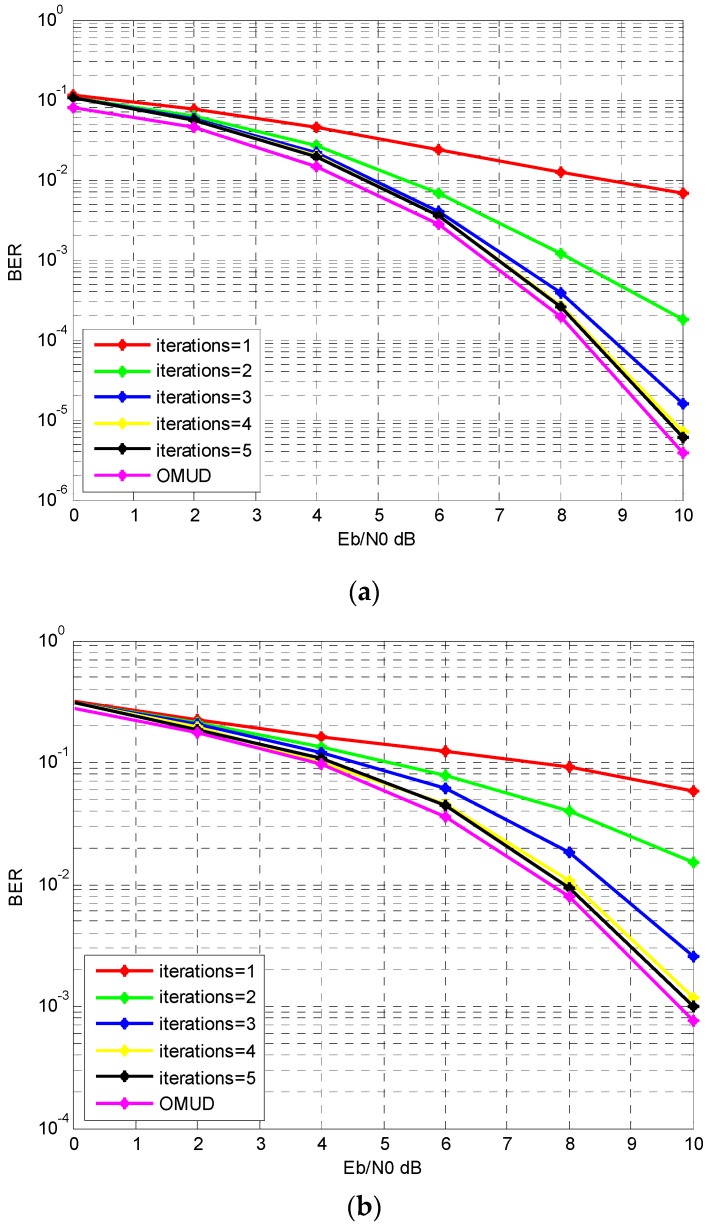
The BER performance with different number of iterations (50 users). (**a**) AWGN; (**b**) IEEE 802.15.4a CM1.

First we assume that there exist 50 users simultaneously in a WSN. For balanced Gold sequence with length 127, the cross correlation value $\rho_{ij}$ can be −0.134, −0.008, and 0.118. At the receiver, assume that all the 50 users have the same power. In Figure 5, the receiver employ the proposed iterative multiuser detection algorithm is demonstrated for the first five iterations. The optimal multiuser detector (OMUD) performance is also shown.

It can be seen in Figure 5 that after four iterations, the BER performance improves little when the iteration continues both for the AWGN and IEEE 802.15.4a CM1 channel. Besides, as a suboptimal multiuser detection algorithm, the proposed iterative multiuser detection algorithm does not converge toward the OMUD performance in this 50 user system. However, we can see that the performance gap between OMUD and the proposed multiuser detector is small after five iterations. For the IEEE 82.15.4a CM1 channel, we can see that there exists a distinct performance loss compare to the AWGN channel. It is mainly because that the S-RAKE receiver does not gather all the paths which results in energy loss.

### 4.2. BER Performances of Different E_b_/N_0_

In this subsection, we consider the 20 and 50 user conditions. The number of iterations for the proposed multiuser detection algorithm is 5. The performance of MF, DEC, MMSE and OMUD are compared.

**Figure 6:**
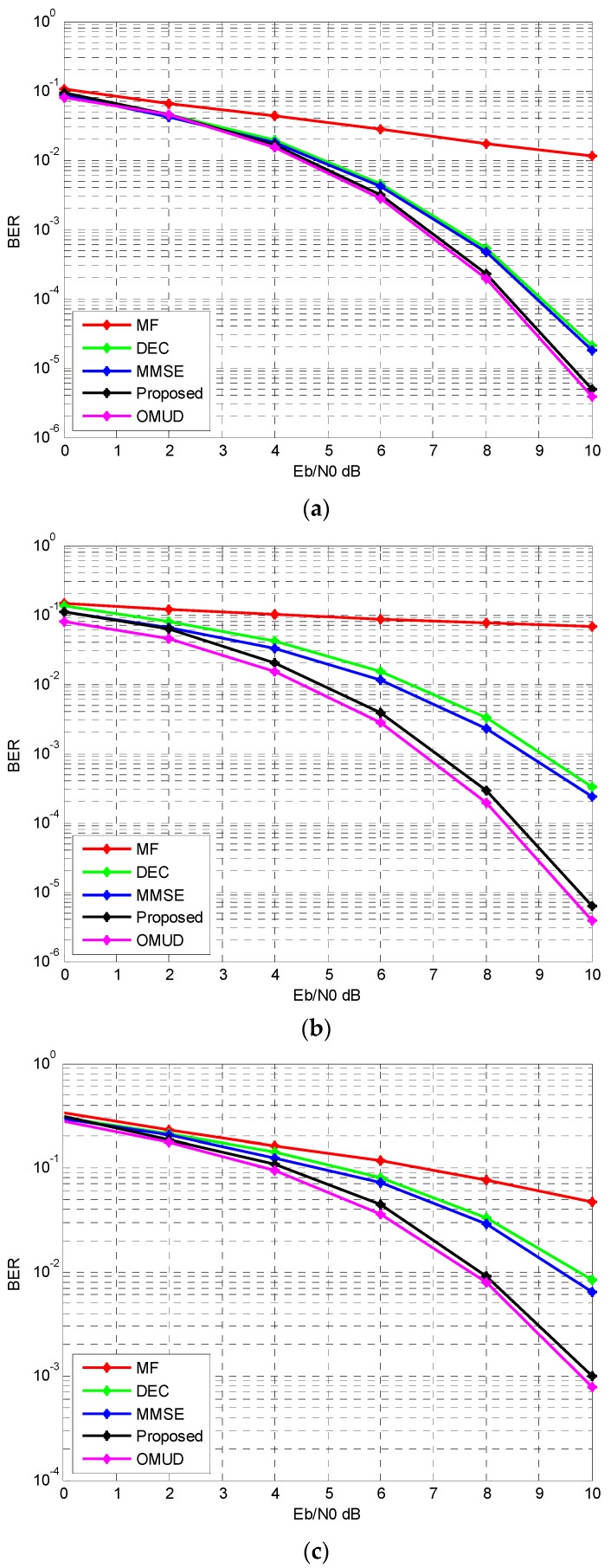
The BER performance with different *E_b_*/*N*_0_. (**a**) AWGN, 20 users; (**b**) AWGN, 50 users; (**c**) IEEE 802.15.4a CM1, 50 users.

Assuming all users have the same power at the receiver, simulation results are illustrated in Figure 6. From the figure it is clear that the BER performance of the proposed iterative multiuser detection method is superior compared with MF, DEC, and MMSE. In 20 user conditions, it even coincides with OMUD. In 50 user conditions, there still exists a considerable gap between the proposed multiuser detection algorithm and OMUD. Besides, it can be seen that for the AWGN channel and the multipath IEEE 802.15.4a channel we can get similar results.

### 4.3. Near-Far Effect Resistance Performance Comparison

**Figure 7:**
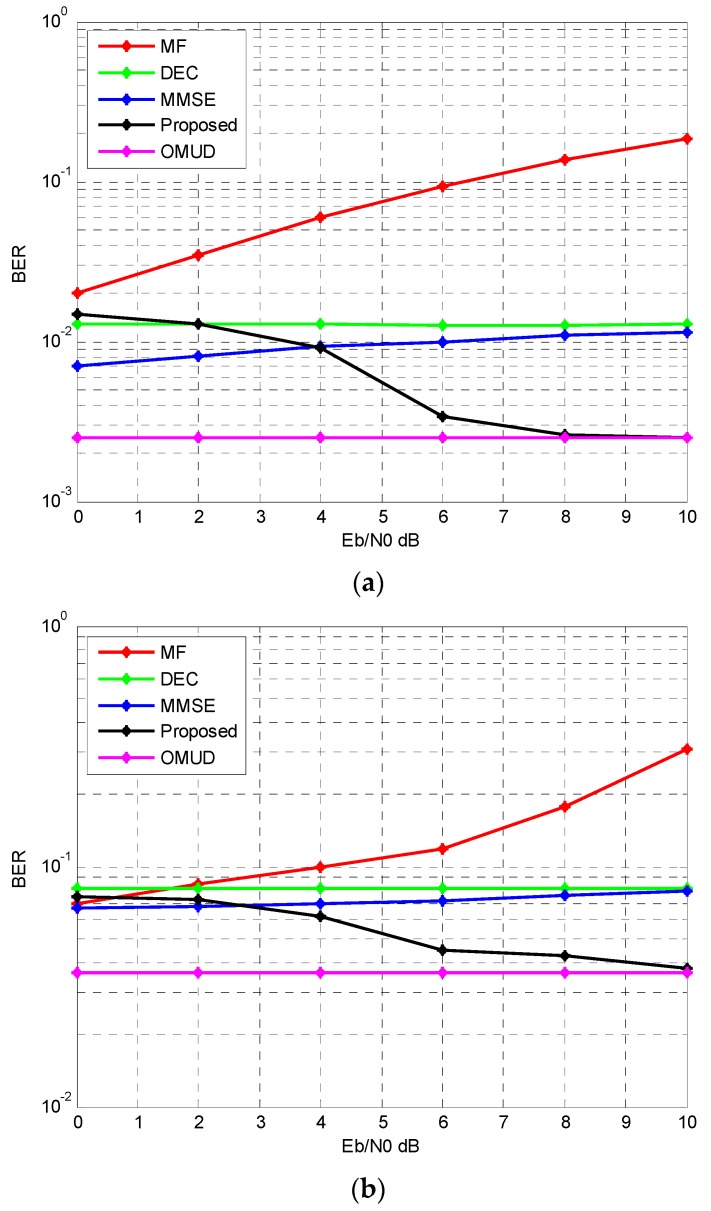
The near-far effect resistance performance (50 users). (**a**) AWGN; (**b**) IEEE 802.15.4a CM1.

In this simulation, these tested multiuser detection algorithm is under the imperfect power control assumption, i.e., the power is not equal at the receiver for each user. Assume a 50 user condition; *E_b_*/*N*_0_ of the first user is 6 dB, while *E_b_*/*N*_0_ of the remaining 49 users changes from 0 dB to 10 dB simultaneously. For the proposed multiuser detection algorithm, 5 iterations are employed. The result is shown in Figure 7.

As shown in Figure 7, for both channels we get similar near-far effect resistance results. In this 50 user system when the SNR of interference user is larger than 4 dB, the BER performance is better than MF, DEC and MMSE; when the SNR of interference user increases up to 10 dB, the BER performance even coincides with OMUD. Besides, the performance of OMUD and DEC remain unchanged when the power of interference users change. While MF and MMSE suffers performance degradation when the power of interference users get stronger. For the proposed multiuser detector, the weak users actually benefit from the strong interference whereas the strong users suffer performance loss from the weak interference. This phenomenon was also observed in other multiuser detection algorithms based on interference cancellation [28,29].

### 4.4. User Capacity Performance Comparison

**Figure 8:**
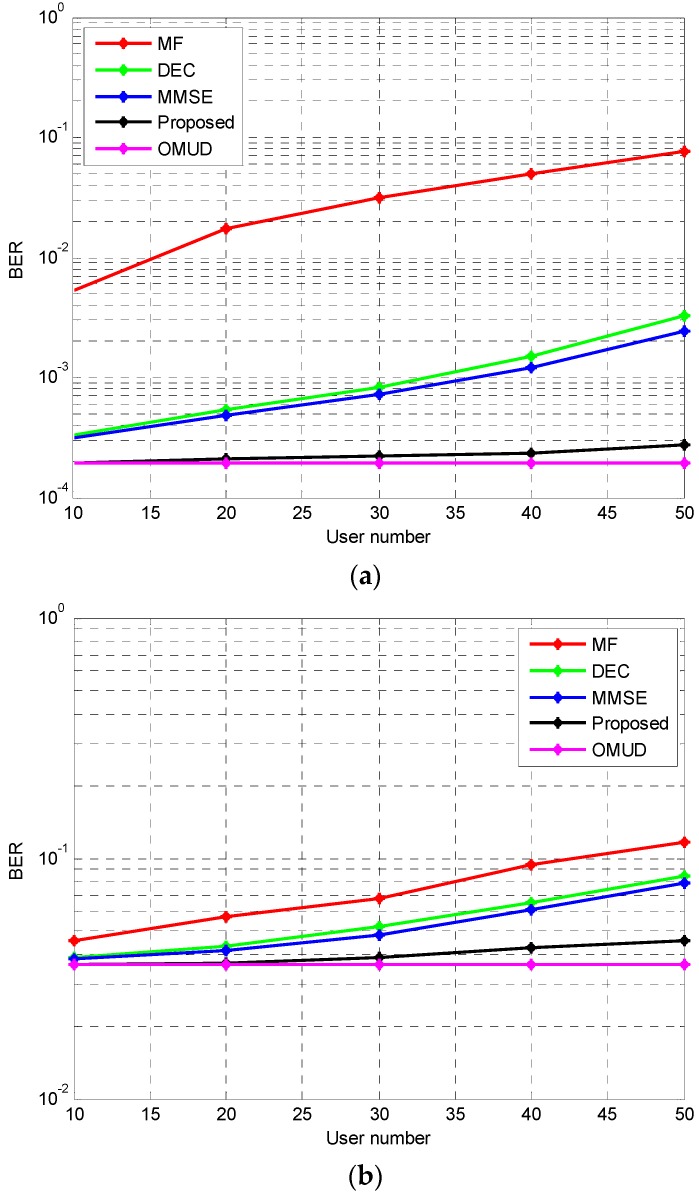
The BER performance with different number of users. (**a**) AWGN; (**b**) IEEE 802.15.4a CM1.

The BER performance curves of these multiuser detection algorithms with different number of users are explored here. In this simulation, *E_b_*/*N*_0_ of all users is 6 dB. Five iterations are simulated for the proposed iterative multiuser detection algorithm. The simulation result is shown in Figure 8.

From Figure 8 we can see that as user number increases, the BER performance of MF, DEC, MMSE and the proposed receiver falls, while the BER performance of the proposed iterative multiuser detection method is much better than MF, DEC, and MMSE when user number ranges from 10 to 50. When user number is smaller than 20, it even coincides with OMUD.

## 5. Conclusions

In this paper, an iterative multiuser detection algorithm based on differential characteristics for DS-UWB systems is explored. In this three stage receiver, the differential characteristics function is calculated; then threshold detection is utilized to get a preliminary classification; the third stage is to further correct the error bits. The above three stage process procedure is iterated to further lower BER. The computational complexity of the proposed receiver is linear to *nK*, where *n* is the number of iterations and *K* is the number of users. Simulation results illustrates that the proposed iterative multiuser detection algorithm convergences after only a few iterations. Furthermore, the BER performance, near-far resistance performance and user capacity performance are superior compared with MF, DEC, and MMSE.

## Figures and Tables

**Table 1:** Simulation parameters.

System	DS-UWB
Modulation mode	BPSK
Pseudo random sequences (PRS)	Balanced Gold sequences
Length of PRS	127
Number of tested bits	50,000,000
Communication channel	AWGN
